# Intravenous administration of anti-inflammatory mesenchymal stem cell spheroids reduces chronic alcohol intake and abolishes binge-drinking

**DOI:** 10.1038/s41598-018-22750-7

**Published:** 2018-03-22

**Authors:** Fernando Ezquer, Paola Morales, María Elena Quintanilla, Daniela Santapau, Carolyne Lespay-Rebolledo, Marcelo Ezquer, Mario Herrera-Marschitz, Yedy Israel

**Affiliations:** 1 Centro de Medicina Regenerativa, Facultad de Medicina Clínica Alemana-Universidad del Desarrollo, Santiago, Chile; 2 Molecular and Clinical Pharmacology Program, Institute of Biomedical Sciences, University of Chile, Santiago, Chile; 3 Department of Neuroscience, Faculty of Medicine, University of Chile, Santiago, Chile

## Abstract

Chronic alcohol intake leads to neuroinflammation and astrocyte dysfunction, proposed to perpetuate alcohol consumption and to promote conditioned relapse-like binge drinking. In the present study, human mesenchymal stem cells (MSCs) were cultured in 3D-conditions to generate MSC-spheroids, which greatly increased MSCs anti-inflammatory ability and reduced cell volume by 90% versus conventionally 2D-cultured MSCs, enabling their intravenous administration and access to the brain. It is shown, in an animal model of chronic ethanol intake and relapse-drinking, that both the intravenous and intra-cerebroventricular administration of a *single* dose of MSC-spheroids inhibited chronic ethanol intake and relapse-like drinking by 80–90%, displaying significant effects over 3–5 weeks. The MSC-spheroid administration fully normalized alcohol-induced neuroinflammation, as shown by a reduced astrocyte activation, and markedly increased the levels of the astrocyte Na-glutamate (GLT-1) transporter. This research suggests that the intravenous administration of MSC-spheroids may constitute an effective new approach for the treatment of alcohol-use disorders.

## Introduction

Alcohol-use disorders constitute a leading cause of morbidity and premature mortality worldwide, accounting for 1 in 10 deaths among working-age adults in the United States^1^. Chronic alcohol intake is also the most common cause of peripheral and central nervous system toxicity^2^.

Several studies in humans and rodents have shown that chronic alcohol consumption leads to an increase in inflammatory cytokines, both in the periphery^3^ and the brain^4,5^. In the brain, alcohol-induced neuroinflammation activates astrocytes and microglia, inducing the secretion of pro-inflammatory cytokines and neurodegeneration^6^. Furthermore, pro-inflammatory conditions increase voluntary alcohol consumption in rodents and humans^7,8^. Research has indicated that alcohol-induced neuroinflammation remains up-regulated for long periods even after discontinuation of alcohol consumption, whereas the peripheral levels return rapidly to normal, indicating the presence of a potent mechanism of auto-perpetuation of neuroinflammation, which could be related to the lack of regulatory T cells in the brain^9,10^. This phenomenon is associated with a marked increase in the risk of relapse in abstinent patients^7,8^.

Neuroinflammation is also associated with the chronic use of other addictive drugs^11,12^ including cocaine, opiates, marijuana and methamphetamine. Drug relapse observed for alcohol and other drugs is causally associated with the existence of high levels of extracellular glutamate, since the administration of N-acetyl cysteine or ceftriaxone which increase the level of the astrocyte Na-glutamate transporter-1 (GLT-1) and remove glutamate from the extracellular space, markedly reduce relapse^13,14^. These studies further suggest the existence of a common mechanism of addictive drug relapse.

Cell therapy based on mesenchymal stem cells (MSCs) is emerging as a clinical option for various diseases in which dysregulation of the immune system is involved^15^. The immunomodulatory potential of MSCs is well documented, indicating several mechanisms for various targets in the immune system^16^. While the possible effect of MSCs on GLT-1 levels has not been addressed, recently in a well-validated animal model of high-alcohol intake^17^, it was reported that the intra-cerebroventricular administration of rat MSCs^18^ or human MSCs that were activated *in vitro* by incubation with TNFα and IFNγ^19^, markedly reduced chronic alcohol intake and relapse-like drinking. Clearly, the intra-cerebroventricular administration is not a preferred route for the treatment of a chronic disease such alcoholism. Although MSCs have been shown to cross the blood-brain barrier when intravenously injected^20,21^, this route is highly inefficient, since, due to their large size; approximately 90% of intravenously administered MSCs are rapidly entrapped in the lungs and other organs causing hemodynamic alterations^22,23^.

Previous studies showed that the three-dimensional aggregation of MSCs in hanging droplets (generating “MSC-spheroids”) greatly reduces their size and enhances their immunomodulatory paracrine secretion, compared with traditional 2D monolayer cultures^15,24^. In MSC-spheroids, mild hypoxic conditioning due to oxygen transport limitation within the 3D cellular structure and increased cell-cell contact have been implicated in the increased paracrine secretion^25,26^. The aggregation of MSCs into 3D spheroids induces a 75% reduction in individual cell volume, as seen when spheroids are disaggregated^15,27^. This size reduction could allow intravenously administered MSC-spheroids to reach the brain.

In this study, we determined whether the intra-cerebroventricular (ICV) or the intravenous administration of human MSC-spheroids to high-alcohol drinker rats reduces neuroinflammation and inhibits chronic alcohol intake and relapse-like drinking.

We also addressed the role of the glutamate transporter GLT-1 in contributing to the effects of MSC-spheroids on ethanol relapse. To assess the possible translational potential of the intravenous administration of MSC-spheroids, the study also determined the distribution of administered MSC-spheroids and the safety of this procedure.

## Results

### MSCs cultured as 3D-spheroid structures increase their anti-inflammatory potential

Human AD-MSCs were isolated from samples of subcutaneous adipose tissue obtained from liposuction aspirates with patient consent and ethical approval. After two sub-cultures, plastic-adherent cells exhibited a robust adipogenic and osteogenic phenotype when stimulated in defined differentiation media (Supplementary Figure 1A). Additionally, after immune-phenotypification plastic-adherent cells showed the presence of surface markers characteristic of MSCs, including CD29, CD13, CD105, CD73, CD90, CD44 and absence of marker characteristics of erythroid precursors (CD235a), endothelial cells (CD31) and hematopoietic cells (CD45) (Supplementary Figure 1B), confirming that isolated cells were MSCs.

**Figure 1:**
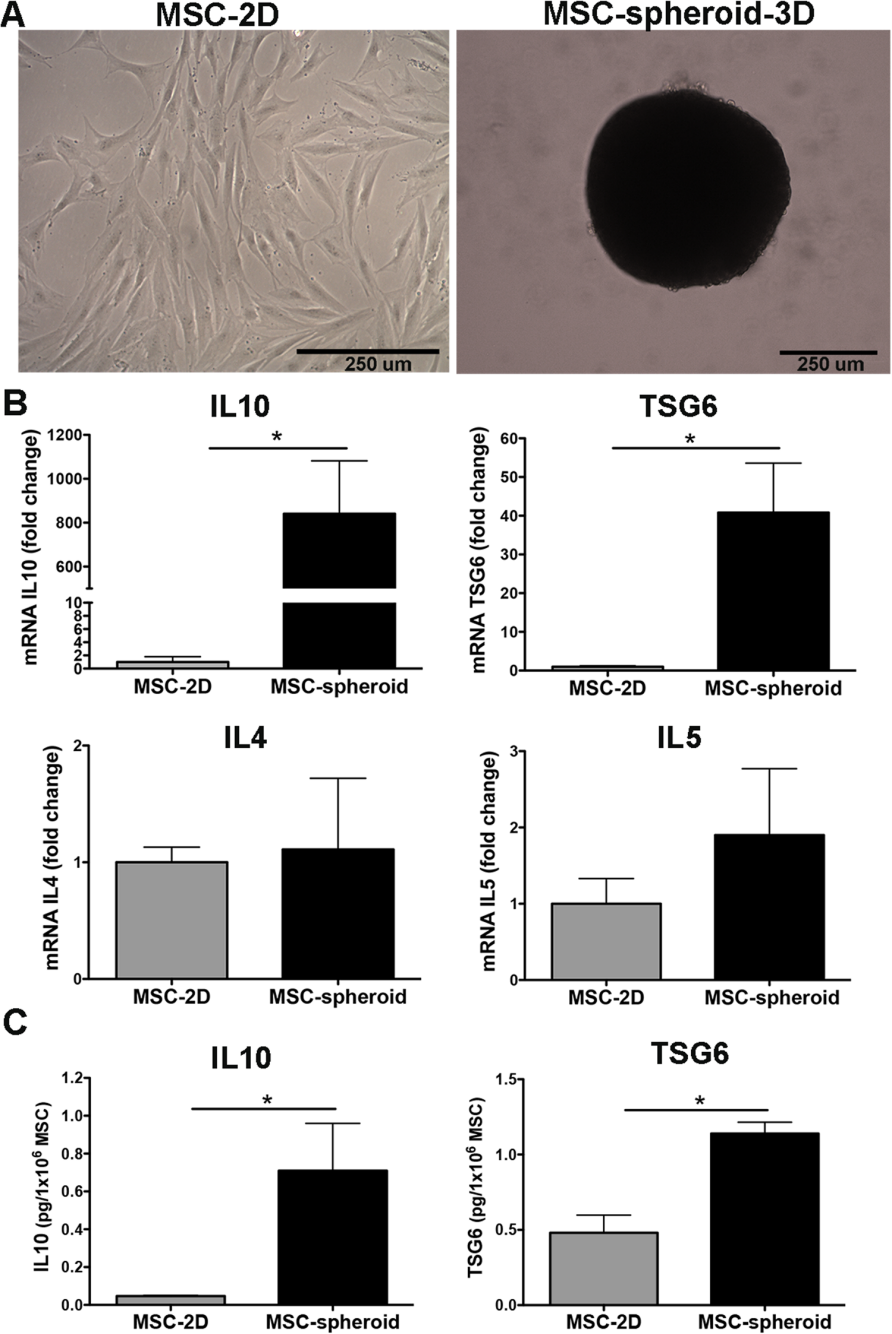
Culture of mesenchymal stem cells into 3D-spheroids increases the production of anti-inflammatory factors. (**A**) Microphotographs of MSCs cultured in conventional 2D culture or *aggregated* into 3D spheroids. Scale bar 250 μm. (**B**) Levels of IL10 and TSG6 mRNA determined by quantitative RT-PCR analysis of 2D-MSCs or 3D MSC-spheroids three days after seeded. Data of each target gene was normalized against the mRNA levels of the housekeeping genes EEF1A1 and RPL13A and presented as fold change of expression in MSC-spheroid versus 2D-MSC. (**C**) Levels of IL10 and TSG6 determined by ELISA in the secretome of 2D-MSCs or 3D MSC-spheroids three days after seeded. Data are presented as pg of IL10 or TSG6 secreted by 1 × 10^6^ MSC-spheroids or 1 × 10^6^ 2D-MSC. Data are shown as mean ± SEM. N = 4 per experimental condition. All determinations *p < 0.05 (see text).

Mesenchymal stem cells were incubated in high-density 3D structures using the hanging drop method (3D-spheroids) and compared to conventional 2D cultures (Fig. 1A). After hanging drop culturing for three days, RT-qPCR was performed to measure the expression level of anti-inflammatory molecules. Compared to conventional 2D-cultured MSCs, MSC-spheroids showed a nearly 900-fold increase in IL10 mRNA levels [Student t test; t: 3. 485, p < 0.025] and a 40-fold increase in TSG6 mRNA levels [Student t test; t: 4.745, p < 0.002] (Fig. 1B), two potent anti-inflammatory molecules. Increased expression of IL10 and TSG6 seems to be specific, since other anti-inflammatory molecules commonly secreted by MSCs, such as IL4 and IL5 were not increased by this procedure (Fig. 1B). Furthermore, the protein levels of IL10 and TSG6 in the secretome of MSC-spheroids also increased [Student t test; t: 3.169, p < 0.025 for IL10 and Student t test; t: 4.742, p < 0.009 for TSG6] compared to 2D-cultured MSCs (Fig. 1C), suggesting that culturing of MSCs into 3D-spheroids improved their anti-inflammatory potential.

### The intra-cerebroventricular administration of MSC-spheroids reduces astrocyte activation and MCP-1 expression

**Figure 2:**
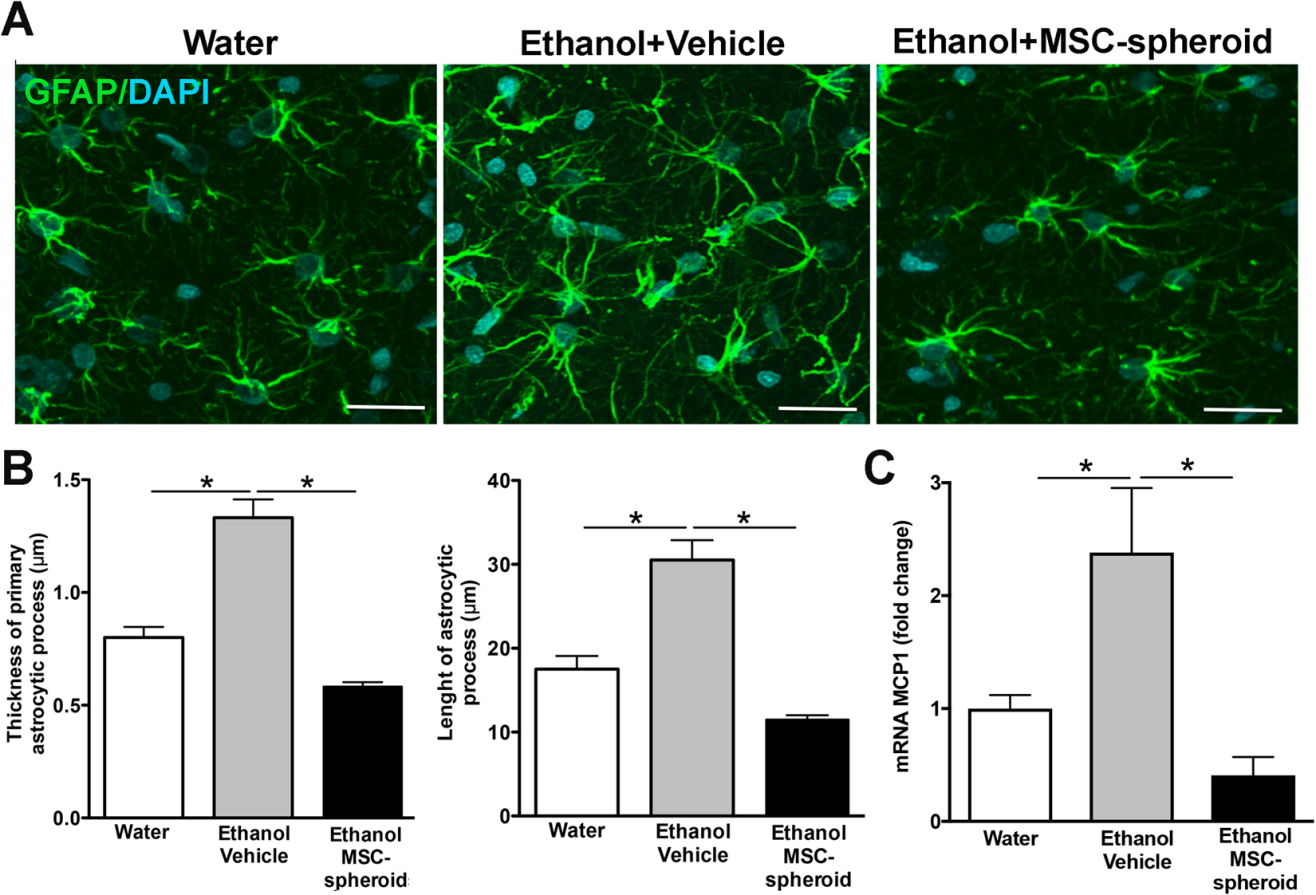
Intra-cerebroventricular administration of MSC-spheroids normalizes both the ethanol-induced activation of hippocampal astrocytes and the increases in MCP-1 levels. (**A**) Confocal microscopy microphotographs of GFAP immunoreactivity was evaluated in hippocampal astrocytes of animals injected intra-cerebroventricularly with 5 × 10^5^ MSC-spheroid or vehicle. Rats that had consumed ethanol for 17 weeks were intra-cerebroventricularly injected with a single dose of 5 × 10^5^ MSC-spheroids or vehicle. After the MSC-spheroid administration the animals remained for one additional week in an ethanol and water free-choice condition, followed by a 2-week ethanol deprivation period and thereafter were allowed a 60-min period of ethanol re-access and euthanized for immunohistochemistry and mRNA studies. Animal consuming only water were used as controls. Scale bar 25 μm. (**B** left) Thickness and (**B** right) length of primary astrocytic process evaluated by confocal microscopy and FIJI image analysis software. (**C**) Level of mRNA of the pro-inflammatory factor MCP-1 in the hippocampus of the same animals, determined by quantitative RT-PCR analysis. Data were normalized against the mRNA levels of the housekeeping genes ß-actin and GAPDH. Data are shown as mean ± SEM. N = 6 per experimental condition. All determinations, *p < 0.05 (see text).

The histologic hallmark of alcohol-induced neuroinflammation is the activation of astrocytes, seen as an enlargement and increased thickness of astrocytic processes assessed by immunoreactivity against GFAP^4^. Astrocyte activation also induces the secretion of several pro-inflammatory molecules, including the monocyte chemoattractant protein-1 (MCP-1). To assess whether MSC-spheroid administration reduced alcohol-induced neuroinflammation, spheroids were disaggregated and 5 × 10^5^ MSC-spheroids were ICV injected. GFAP immunoreactivity was evaluated in hippocampus samples obtained 21 days after MSC-spheroid or vehicle administration. Animals that were never exposed to ethanol (water group) were used as controls. Chronic alcohol consumption induced a significant increase in the thickness [one-way ANOVA; *F*_(2,394)_ = 31.86, *p* < 0.0001] and length [one-way ANOVA; *F*_(2,584)_ = 27.12, *p* < 0.0001] of primary astrocytic processes, also inducing a significant increase in the MCP-1 mRNA level in the hippocampus [one-way ANOVA; *F*_(2,14)_ = 5.988, *p* < 0.01] (Fig. 2A,B and C). The administration of MSC-spheroids fully reversed the changes induced by chronic ethanol intake, restoring to normal levels the hippocampal astrocyte GFAP and MCP-1 (Fig. 2A,B and C), thus indicating a strong anti-inflammatory effect of MSC-spheroids.

### Intra-cerebroventricular (ICV) administration of a single dose of MSC-spheroids greatly reduces chronic ethanol intake and relapse-like drinking after ethanol deprivation

**Figure 3:**
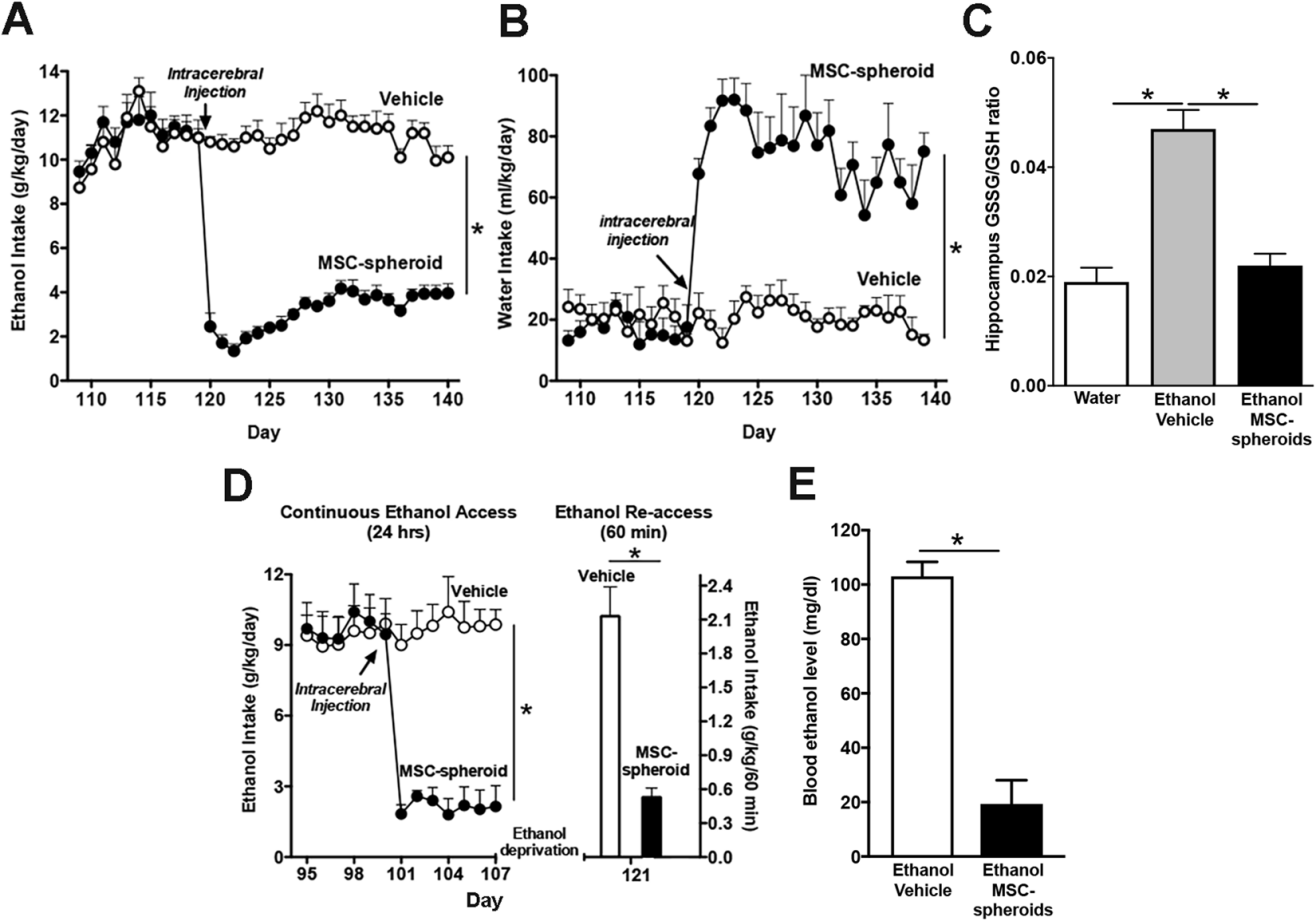
Intra-cerebroventricular administration of MSC-spheroids inhibits chronic ethanol intake and relapse-like ethanol intake in the (ADE) ethanol post-deprivation condition and normalizes oxidative stress (GSSG/GSH). (**A**) Voluntary ethanol intake of animals that had consumed ethanol for 17 weeks and were injected with a single dose of 5 × 10^5^ MSC-spheroid or vehicle into the left cerebral ventricle. Data are expressed as g of pure ethanol consumed/kg body weight/day. (**B**) Voluntary water intake in the same animals of figure A. Data are expressed as ml water consumed/kg body weight/day. A large proportion of the calories in the alcohol group is consumed in liquid form, while the water (chow) group consumes all their calories from a solid dry diet throughout the study. Their water consumption it is not indicated (nor it was determined) (**C**) GSSG/GSH ratio in hippocampus samples obtained twenty-two days after MSC-spheroid or vehicle administration. Animals consuming only water were used as controls. (**D**) Relapse-like drinking in animals that had consumed ethanol for 14 weeks and were injected with a single dose of 5 × 10^5^ MSC-spheroid or vehicle into the left cerebral ventricle. Voluntary ethanol intake was determined after a 60-minute ethanol re-access, following a 14-day ethanol deprivation period. Ethanol intake during the 60-minute re-access is expressed as g of pure ethanol consumed/kg body weight/60 minutes. (**E**) Blood ethanol level attained by animals in Figure D after the 60-minute relapse-like ethanol consumption. Data are shown as mean ± SEM. N = 6 per experimental condition. Repeated measures analyses were conducted for Fig. 3A and D; left side and Fig. 3B. All determinations, *p < 0.05 (see text).

To evaluate the effect of MSC-spheroids on chronic ethanol intake, 5 × 10^5^ disaggregated MSC-spheroids were ICV injected into rats that had ingested ethanol under free choice of 10% ethanol *versus* tap water for 17 weeks. Prior to MSC-spheroid administration rats consumed 10–12 g ethanol/kg/day. An 88% reduction in ethanol intake was observed 24 hours after MSC-spheroid administration compared to vehicle-treated rats [Student t test; t: 10.29 p < 0.0001] (Fig. 3A). An inhibitory effect on chronic ethanol intake was still observed after three weeks, time at which 60% of the maximum inhibitory effect was present [two-way ANOVA; *F*_*treatment* (1,210)_ = 2427.0, *p* < 0.0001] (Fig. 3A). The reduction in ethanol intake was clearly correlated with an increased in water intake (Fig. 3B) [two-way ANOVA; *F*_*treatment* (1,200)_ = 466.4.3, *p* < 0.0001]. Therefore, the hydric balance was maintained. Three weeks after the MSC injection, the hippocampal GSSG/GSH ratio was 150% higher in chronic ethanol-drinking animals treated with vehicle versus ethanol naïve animals, an increase that was fully normalized by MSC-spheroid administration [one-way ANOVA; *F*_(2,18)_ = 29.93, *p* < 0.0001] (Fig. 3C).

To evaluate whether MSC-spheroids could also inhibit relapse-like drinking, a new group of rats that had been drinking 10% ethanol for 14 weeks was ICV injected with a single dose of 5 × 10^5^ MSC-spheroids or vehicle. Voluntary ethanol intake was evaluated for seven days after MSC-spheroids administration and thereafter, a 14-day withdrawal period was imposed to induce the alcohol deprivation effect, followed by a 60-minute ethanol re-access. According with the above data, MSC-spheroid administration induced a rapid reduction in chronic ethanol intake [two-way ANOVA; *F*_*treatment* (1,104)_ = 109.9, *p* < 0.0001] (Fig. 3D left), but also a 75–80% reduction of alcohol-relapse drinking (binge drinking) compared to vehicle-treated animals [Student t test; t: 7. 347, p < 0.0003] (Fig. 3D right).

As expected, the reduction of ethanol consumption in the 60-minute ethanol re-access observed in MSC-spheroids-treated rats correlated with a marked 80% reduction in blood ethanol levels to 20 mg/dl, while vehicle-treated rats showed intoxicating blood ethanol levels, slightly above 100 mg/dl [Student t test; t: 6. 81, p < 0.0005] (Fig. 3E).

### Intravenous administration of MSC-spheroids reduces chronic ethanol intake and relapse-like drinking to the same level achieved by the intra-cerebroventricular route

Researchers have consistently reported that, due to their large size, when 2D-cultured MSCs are administered by the intravenous route, over 90% of administered cells are trapped in the lungs and other organs^23,28^. Thus, the number of MSCs that could reach the brain by the intravenous route is extremely low. As indicated, the culture of MSCs in 3D-spheroids structures significantly reduces MSC size^27^, which could prevent MSC lodging in several organs, increasing the number of cells that could reach the brain.

To evaluate whether the intravenous administration of MSC-spheroids also has a therapeutic potential in the reduction of ethanol intake, rats that had consumed ethanol for 12 weeks were intravenously injected with a single dose of 1 × 10^6^ MSC-spheroids or vehicle.

**Figure 4:**
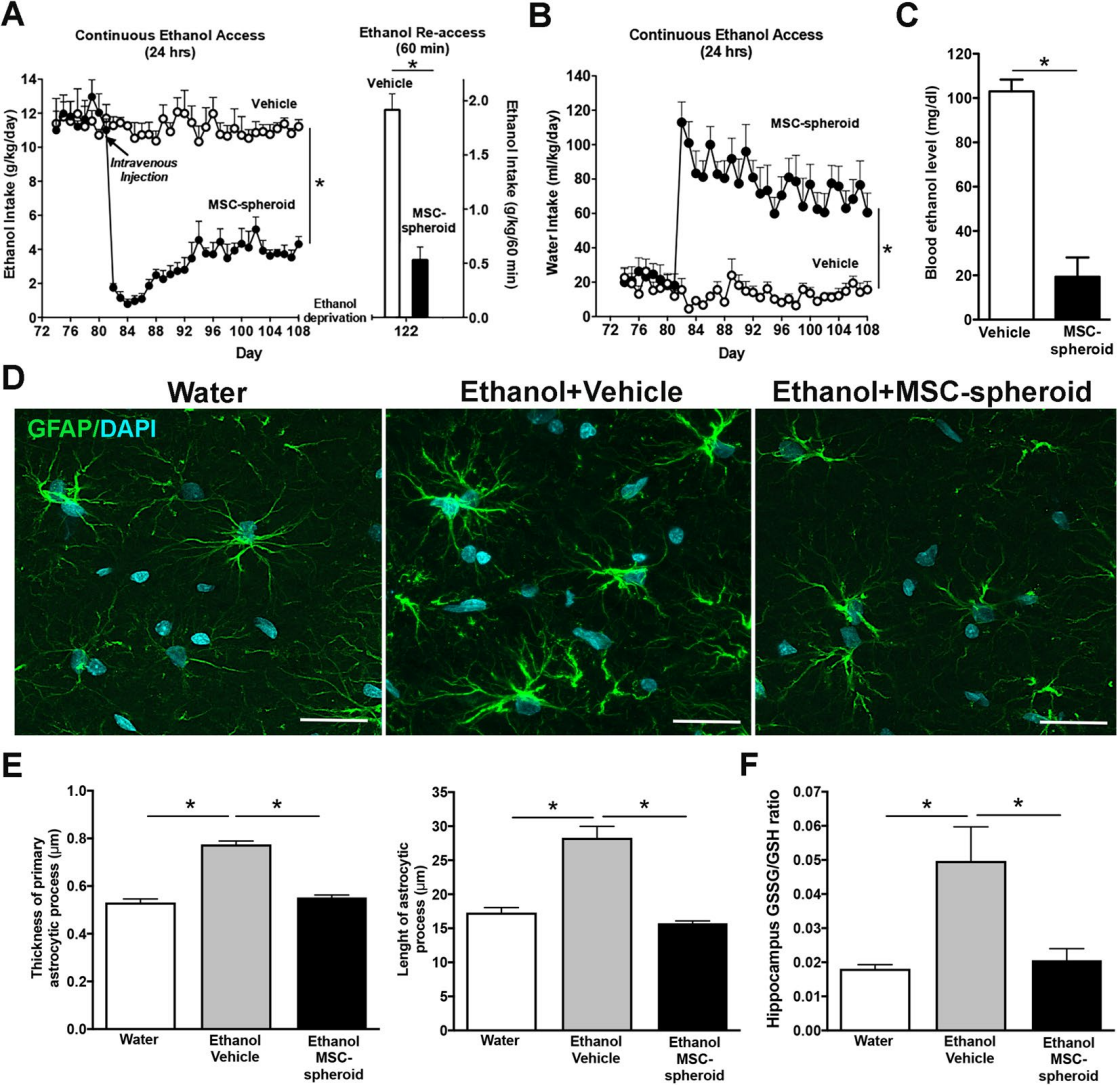
Intravenous administration of MSC-spheroids inhibits chronic ethanol intake and relapse-like ethanol intake in the ethanol post-deprivation condition (ADE), and normalizes astrocyte (GFAP) activation and oxidative stress (GSSG/GSH ratio): (**A** left) Voluntary ethanol intake of animals that had consumed ethanol for 12 weeks and were intravenously injected with a single dose of 1 × 10^6^ MSC-spheroids or vehicle. Ethanol intake is expressed as g of pure ethanol consumed/kg body weight/day. (**A** right) Rats that had consumed ethanol for 12 weeks were injected with a single dose of 1 × 10^6^ MSC-spheroids or vehicle. After the intravenous MSC-spheroid administration the animals remained for four weeks in the ethanol and water free-choice condition, followed by a 2-week ethanol deprivation period. Relapse drinking after the deprivation period was determined by allowing animals a 60-minute ethanol re-access. Ethanol intake during the 60-minute re-access is expressed as g of pure ethanol consumed/kg body weight/60 minutes. (**B**) Voluntary water intake in the same animals of figure A. Data are expressed as ml of water consumed/kg body weight/day. Repeated measures analyses were conducted for Fig. 4A,B. (**C**) Blood ethanol level evaluated after the 60-minute relapse-like ethanol consumption. (**D**) Confocal microscopy microphotographs of GFAP immunoreactivity evaluated in hippocampal astrocytes of animals intravenously injected with 1 × 10^6^ MSC-spheroid or vehicle, after the 60-minute ethanol re-access. Animals consuming only water were used as control. Scale bar 25 μm. (**E** left) Thickness and (**E** right) length of primary astrocytic process evaluated by confocal microscopy. (**F**) GSSG/GSH ratio in hippocampus samples evaluated after the 60-minute relapse-like ethanol consumption. Data are shown as mean ± SEM. N = 6 per experimental condition. All determinations, *p < 0.05 (see text).

We observed a marked reduction, initially a 93% reduction, in voluntary ethanol intake [2-way ANOVA; F_treatment (1,350)_ = 1100, p < 0.0001], and a significant increase in water intake [2-way ANOVA; F_treatment (1,200)_ = 466.4, p < 0.0001] in MSC-spheroids treated animals compared with vehicle-treated rats, which lasted over four weeks (Fig. 4A left and B). The reduction of ethanol intake observed after MSC-spheroid administration by the intravenous route was comparable to the inhibition achieved by MSC-spheroid administration by the ICV route (88% reduction by the ICV route *vs*. 93% reduction by the intravenous route).Compared to vehicle treated rats, the intravenous administration of MSC-spheroids also reduced by 80% the relapse-like binge drinking after a 14-day alcohol deprivation period [Student t test; t: 7.347, p < 0.0001] (Fig. 4A right) and decreased blood ethanol levels after the 60-minute ethanol re-access [Student t test; t: 4.096, p < 0.002] (Fig. 4C).

As for the ICV administration of MSC-spheroids, the administration of MSC-spheroids by the intravenous route also reduced the increase in the thickness [one-way ANOVA; F_(2, 569)_ = 105.0; p < 0.0001] and length [one-way ANOVA; F_(2,1221)_ = 66.49 p < 0.0001] of primary astrocytic processes in the hippocampus observed in ethanol-vehicle group compared with the water group (Fig. 4D,E) and also normalized the hippocampal increase in the GSSG/GSH ratio observed in ethanol-vehicle group compared with the water group [one-way ANOVA; F_(2,17)_ = 4.54, p < 0.0001] (Fig. 4F), indicating that MSC-spheroids administered via the intravenous route also effectively reduce the ethanol-induced neuroinflammation and oxidative stress. Animal general behavioral and safety issues are subsequently indicated.

### Intravenous administration of MSC-spheroids increases the astrocyte Na-glutamate transporter-1 (GLT-1) levels

**Figure 5:**
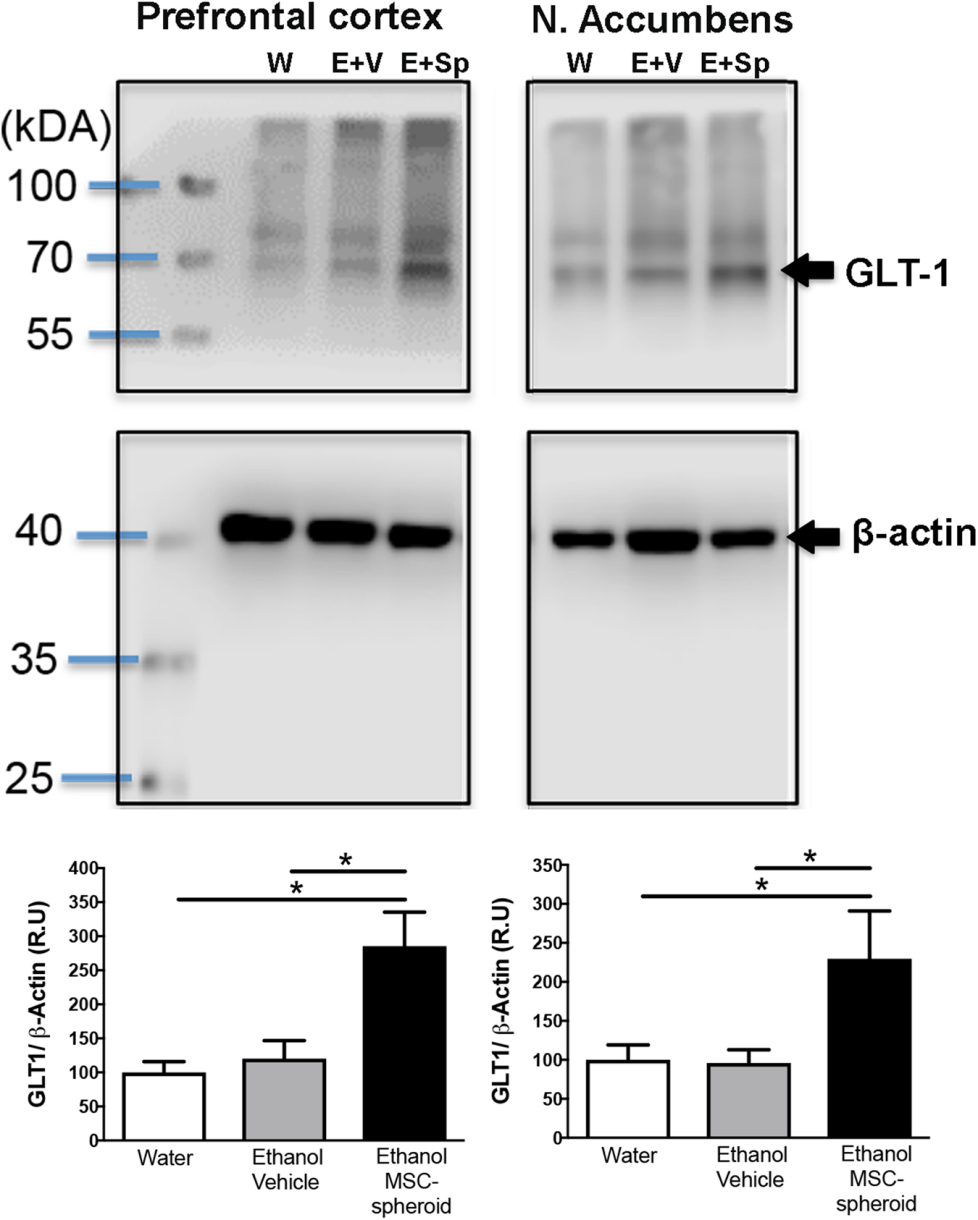
Intravenous administration of MSC-spheroids increases GLT-1 levels. GLT-1 level in prefrontal cortex and nucleus accumbens of rats that had consumed ethanol for 12 weeks and were intravenously injected with a single dose of 1 × 10^6^ MSC-spheroids or vehicle. GLT-1 levels were evaluated by Western blot analysis six weeks after MSC-spheroid or vehicle administration. After the MSC-spheroid administration the animals remained for four weeks in the ethanol and water free-choice condition, followed by a 2-week ethanol deprivation period and a 60-minute ethanol re-access. Animals consuming only water were used as untreated controls. Data are presented as percentage ratios of GLT-1/β-actin, relative to control levels. Data are shown as mean ± SEM. Immunoblots shown are representative of N = 6 per experimental condition. *p < 0.05. W: water; E + V: Ethanol + vehicle; E + Sp: Ethanol + MSC-spheroid.

The levels of the Na-glutamate transporter-1 (GLT-1) were measured by Western blot in animals that had consumed ethanol for 12 weeks and were intravenously injected with a single dose of 1 × 10^6^ MSC-spheroids or vehicle. Following the intravenous MSC-spheroid injection, the rats continued under the ethanol and water free-choice condition for four weeks, while on the subsequent two weeks animals were deprived of ethanol and thereafter were allowed a 60-min period of ethanol re-access. Subsequently, samples of prefrontal cortex and nucleus accumbens were processed for Western blot analyses (*i.e*., six weeks after the administration of the MSC-spheroids). In line with the anti-inflammatory effect and the inhibitory effect on alcohol-relapse of MSCs, the intravenous administration of MSC-spheroids increased the levels of GLT-1 by 180% in prefrontal cortex [one-way ANOVA; F_**(2,13)**_ = 8.52, *p* < 0.005] (Fig. 5) and by 120% in nucleus accumbens [one-way ANOVA; F_**(2,14)**_ = 4.5, *p* < 0.05] (Fig. 5), compared with rats drinking only water or vehicle treated rats.

### Intravenously injected MSC-spheroids efficiently reach the brain

**Figure 6:**
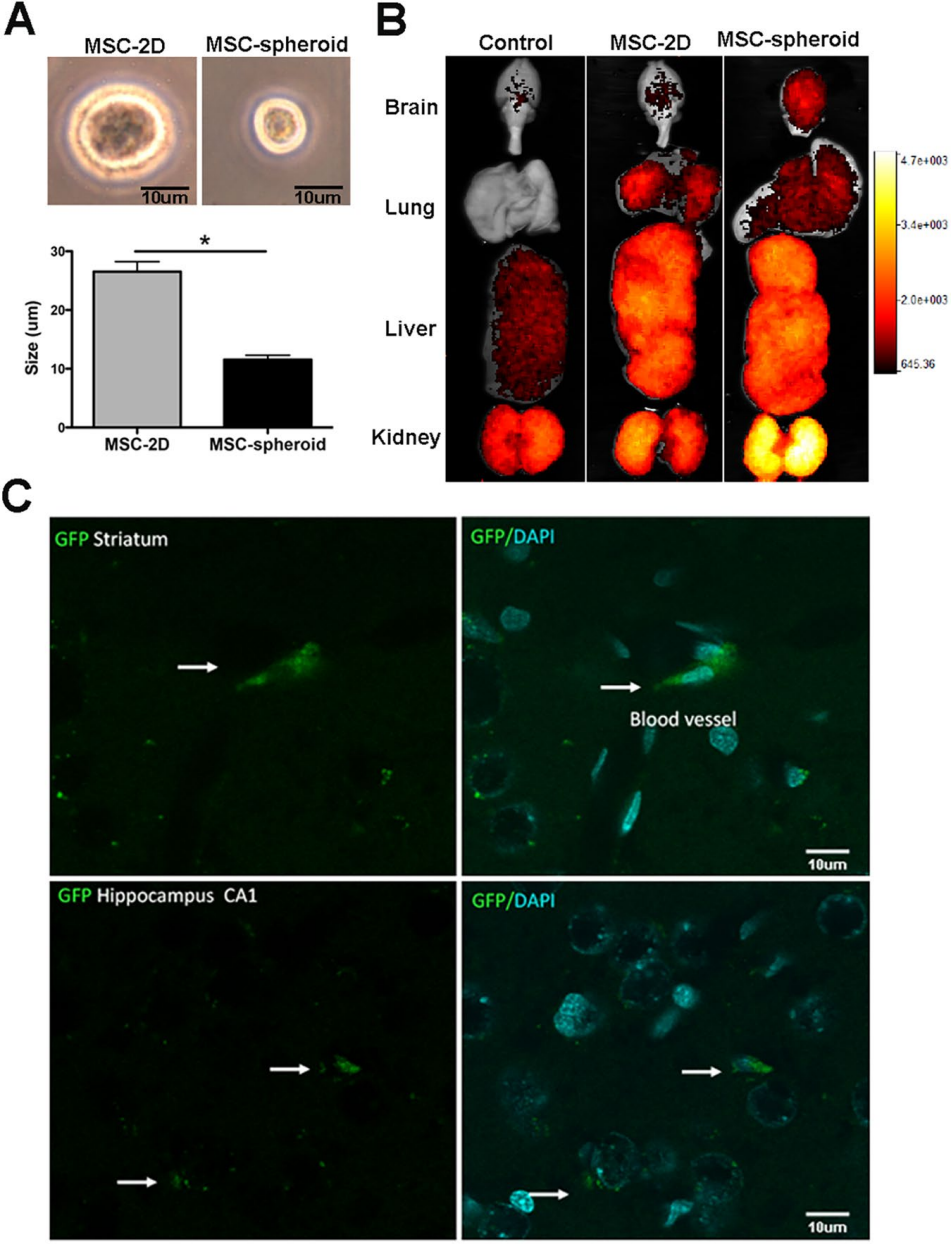
Intravenously injected MSC-spheroids efficiently reach the brain while 2D-MSCs remain logged in other organs. (**A**) MSC size evaluated by optic microscopy and flow cytometry after trypsinization of human MSCs cultured for three days in conventional 2D cultures or as 3D spheroids. (**B**) DiR fluorescence of brain, lungs, liver and kidneys of animals that had consumed ethanol for 12 weeks, 24-hours after they were intravenously injected with a single dose of 1 × 10^6^ DiR and GFP-labeled MSC-spheroids; DiR and GFP-labeled 2D-cultured MSCs or vehicle. DiR fluorescence was evaluated using the MS FX PRO image system. (**C**) Confocal microscopy microphotographs showing presence of MSC-spheroids in brain blood vessels and in the brain parenchyma (white arrows). Since GFP-2D-MSCs were not found in the regions inspected in the brain, only GFP-3D-spheroid microphotographs are shown. Scale bar 10 μm. Images are representative of three animals per experimental condition. *p < 0.05.

The difference in cell size between MSCs in conventional 2D-cultures and 3D-spheroids was determined. Three days after seeding, MSCs were trypsinized and cell size was measured by optic microscopy and flow cytometry. As has been reported^27^, we observed a 50–60% reduction in cell diameter [Student t test; t: 8.013, p < 0.0013]; thus, MSC-spheroids had a 90% smaller volume than 2D-cultured MSCs (Fig. 6A). To evaluate whether intravenously injected MSC-spheroids reached the brain, animals that had consumed ethanol for 12 weeks were intravenously injected with a single dose of 1 × 10^6^ 2D-cultured MSCs labeled with DiR and GFP; a single dose of 1 × 10^6^ MSC-spheroids labeled with DiR and GFP; or vehicle. Twenty-four hours after MSC administration, animals were perfused with PBS, the organs were removed, and the presence of MSCs in different organs was evaluated using the MS FX PRO image system, which detects the DiR signal. As expected, intravenously administered 2D-cultured MSCs were mainly trapped in the lungs and liver with few cells reaching the brain (Fig. 6B). Conversely, after intravenous administration of MSC-spheroids, fewer cells were trapped in the lungs while a marked increase in MSC distribution to brain, liver and kidneys was observed (Fig. 6B). The localization of MSC-spheroids in the brain was also confirmed by the presence of GFP positive cells in brain sections. In MSC-spheroid-treated rats, GFP-MSCs were seen adhered to brain blood vessels and were also present in the brain parenchyma compared to the brains of 2D-MSC treated rats in which GFP-MSCs were not found (Fig. 6C). Images are representative of 3 animals per experimental condition.

### Intravenous administration of MSC-spheroid is not associated with adverse events

**Figure 7:**
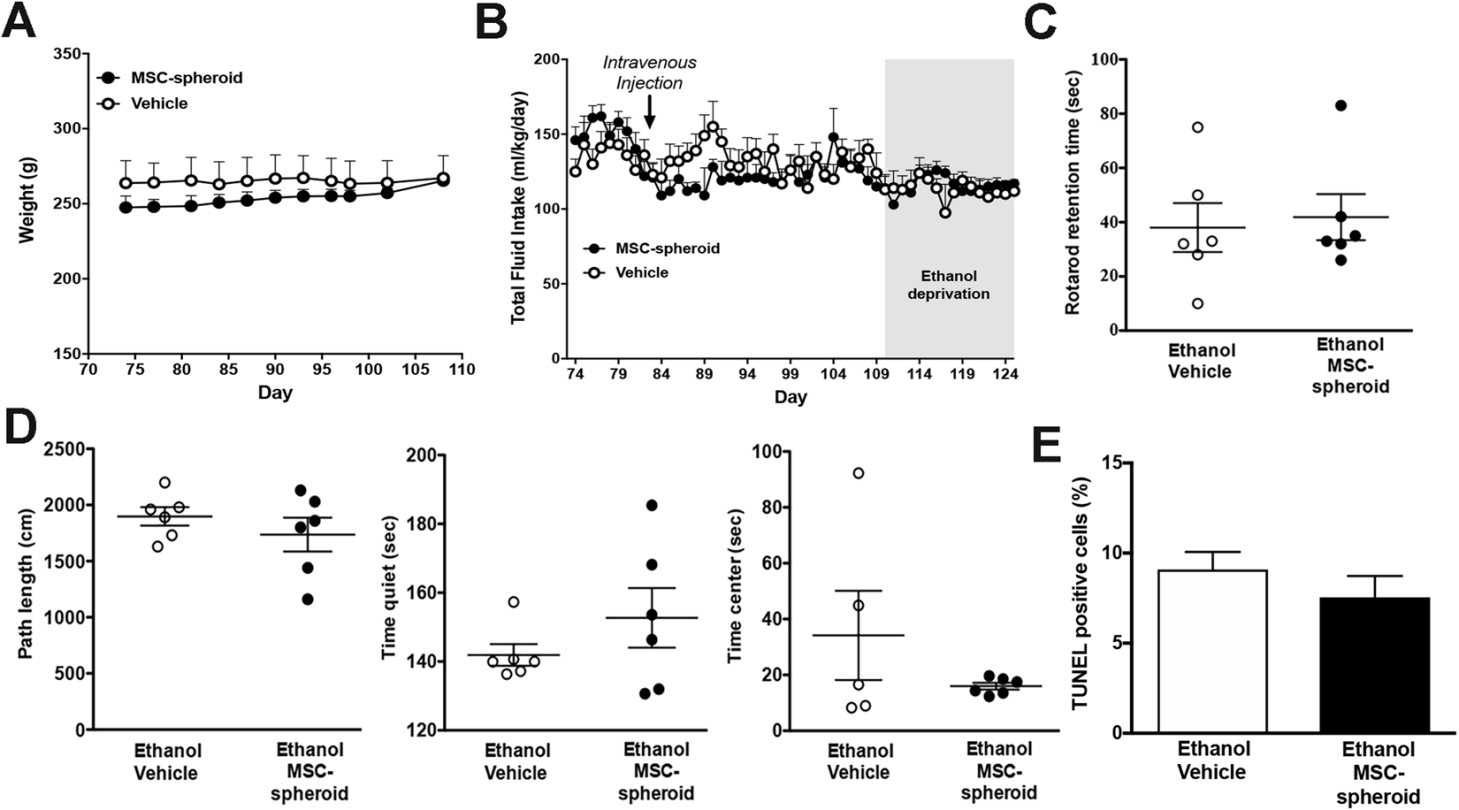
Intravenous administration of MSC-spheroids is not associated to adverse reactions. (**A**) Body weight and (**B**) total fluid intake of animals that had consumed ethanol for 12 weeks and were intravenously injected with a single dose of 1 × 10^6^ MSC-spheroids or vehicle. Total fluid intake is expresses as ml of water + water in ethanol consumed/kg body weight/day. (**C**) Motor coordination evaluated forty days after MSC-spheroid or vehicle administration measuring the retention time using the rotarod test. (**D**) Exploratory behavior examined in a circular arena (open field) forty days after MSC-spheroid administration measuring locomotion, time spent without motion and time spent in the center of the arena. (**E**) Apoptotic rate in the hippocampus evaluated in brain sections by the TUNEL technique. Data are presented as percentage of TUNEL positive cells/DAPI/mm^2^. The administration of MSC-spheroids to alcohol consuming animals did not modify the parameters assessed. Data are shown as mean ± SEM. N = 6 per experimental condition.

Intravenously administered MSC-spheroids showed a broad systemic and brain distribution in alcoholic rats. Therefore, we evaluated if MSC-spheroid administration was associated with any systemic adverse events. No differences in general parameters of wellbeing, including weight gain [2-way ANOVA; F_treatment (1,200)_ = 0.35, p = 0.54; N.S.] or total fluid intake (water + ethanol solutions) [2-way ANOVA; F_treatment (1,56)_ = 2.81, p = 0.09; N.S.] between chronic ethanol consuming rats treated with MSC-spheroids and those treated with vehicle were found (Fig. 7A,B). Additionally, no differences were observed between MSC-spheroid treated rats and vehicle-treated rats in motor coordination [Student t test; t: 0.3087, p = 0.76 N.S.] (Fig. 7C) and in anxiety in an open field test [Student t test, p > 0.05 N.S.] (Fig. 7D). Specific anxiety measures were not different: Open field path length: t = 0.9426, p = 0.368; Time quiet: t = 1.167, p = 0.2702; Time center: t = 1.255, p = 0.2410. Location of MSC-spheroids to brain parenchyma was not associated with an increase in the apoptosis of brain cells [Student t test; t: 1.004 p = 0.32 N.S.] (Fig. 7E). Furthermore, no differences were observed between the experimental groups in plasma levels of markers associated with hepatic damage (AST and ALT), hepatic function (direct bilirubin and total bilirubin) and renal function (creatinine and BUN) [Student t test, p > 0.05 N.S.] (Supplementary Table 1), suggesting that the intravenous administration of MSC-spheroids is safe.

## Discussion

Cumulative evidence indicates that the neuroimmune system and the induction of neuroinflammation contribute to drug abuse and dependence, including alcoholism^11,12^. Postmortem studies show that the level of the pro-inflammatory cytokine MCP-1, a chemokine released following TLR4 activation, and the activity of astrocytes and microglia in hippocampus are elevated in alcoholics compared with non-alcoholic controls^29^. Pro-inflammatory cytokines such as IL1β and NFκB activation are increased in the brain of rats fed alcohol-containing liquid diets^30^, thus linking neuroinflamation to alcohol intake. Accordingly, animals lacking specific innate immune related genes or treated with TLR4 inhibitors, which prevent astrocyte pro-inflammatory responses, showed reduced preference for alcohol drinking^7,31^, while mice that lack MCP-1 or its receptor, display a reduced preference for alcohol^32^.

Marked increases in brain reactive oxygen species (ROS) have been reported following chronic ethanol intake in rodents^33^. ROS are known to inactivate IkB, the NFkB inhibitor, allowing the entrance of the NFkB p65 moiety into the nucleus, thus activating the synthesis of pro-inflammatory cytokines, including TNF-α^34^. A vicious cycle develops since TNF-α further uncouples mitochondria, inducing superoxide ion generation^34^ and the production of additional ROS.

While in the present studies is not possible to firmly conclude that the reduction in ethanol intake induced by MSC-spheroids *followed* the reduction in neuroinflammation, the fact that inducing neuroinflammation by lipopolisaccharde administration leads to *increases* in ethanol intake^7^ strongly suggests that the reduction in alcohol-induced neuroinfammation by MSC led to the reduction in alcohol intake.

It is noted that MSCs are non-immunogenic due to their low expression of antigen-presenting molecules^35^ which limits the ability of MSCs to trigger an unwanted allogeneic response when transferred into an un-matched recipient, allowing xenotransplantation without the need of immunosuppression^36–38^.

A possible neurochemical mechanism for the regulation of cued drinking behavior is an over-activation of the glutamatergic transmission^39,40^. A hyper-glutamatergic state would drive drinking behavior, as it activates the limbic dopamine system, potentiating the cued reward system^41^. In the present study, increases in GLT-1 *protein* levels were not observed in the brain of rats that had chronically consumed ethanol for 12 weeks and while deprived of ethanol for two weeks. However, in the latter a marked increase in oxidative stress is seen as shown by an elevated GSSH/GSH ratio. Oxidation of adjacent cysteine residues in GLT-1 greatly reduces the activity of this transporter without altering the GLT-1 protein level^42^, which was detected by western blot analysis. Thus, an excess of glutamate likely exists in the synaptic region in oxidative stress conditions. A normalization of the GSSG/GSH ratio as well as the ant-inflammatory effect of MSC-spheroids is expected to increase the removal of glutamate by elevating GLT-1 protein. Present studies are in line with reports that increases in GLT-1 levels markedly inhibit the relapse of many addictive drugs^14^.

The anti-inflammatory and antioxidant effects of MSCs are well documented in animal models of stroke, cerebral ischemia and traumatic brain injury, where markers of neuroinflammation and oxidative stress are reduced by MSCs^43,44^. The present study used MSC-spheroids, since culturing MSCs into 3D structures greatly improves the generation and the secretion of the anti-inflammatory cytokines IL10 and TSG6, which counter the inflammatory effects of TNFα and IL1β, inhibiting NF-κB activation^45^. These results are in line with observations of other groups who observed that the culture of MSCs in 3D-spheroids greatly improves the paracrine immunomodulatory activity of these cells^24,46^. We showed that a single ICV injection of human MSC-spheroids induced a 90% reduction in chronic alcohol intake. The effect was long-lasting, as 60% of the maximum inhibitory effect was retained three weeks after MSC-spheroids administration, suggesting a potent therapeutic effect of the cells. In line with the neuroinflammation-ROS potentiation^30^ and the powerful antioxidant potential of MSCs, the ICV administration of MSC-spheroids also fully inhibited the ethanol-induced increases in the GSSG/GSH ratio in the hippocampus.

Binge drinking (≥5 drinks on one occasion for men and ≥4 drinks on one occasion for women), is a common form of relapse behavior. Binge drinking, as well as chronic ethanol intake at moderate levels, induce neuroinflammation, partly due to increases in gut permeability via the generation of gut acetaldehyde disrupting intestinal tight junctions, thus allowing the gut biome bacterial endotoxins to enter the portal circulation^47^. Released LPS potentiates the secretion of pro-inflammatory cytokines by the liver and the effect of these cytokines is expressed across intracerebrally via TNF-α receptors to activate brain neuroimmune signaling that persists for long periods^48^. This pro-inflammatory mechanism also leads to increases in ROS^4^.

In the present study, rats that had consumed ethanol chronically for 2 to 3 months, without overt intoxication were administered MSC-spheroids. Subsequently, a two-week period of alcohol deprivation followed by 60-minute alcohol re-access (ADE) was applied, a condition after which highly intoxicating amounts of ethanol are consumed, akin to binge-drinking. This paradigm has been considered to be the primary model to assess relapse-like behavior in rats^49,50^. Using this animal model, the study showed that the administration of a single dose of MSC-spheroids, whether ICV or intravenously, greatly reduced binge drinking.

The histological and biochemical analyses in this report concentrated on the hippocampus; the brain region most pathologically affected by chronic ethanol intake, including a prolonged ethanol intake without binge drinking^51,52^. In this brain area, ethanol activates microglia and astrocytes via TLR4, which can be evidenced by specific morphological changes including increasing the thickness and length of primary processes (i.e., astrocytosis)^51,53^. This activation promotes the release of pro-inflammatory cytokines, chemokines and ROS which in turn, promote neuronal death in hippocampus and other brain regions^54^. Here, it was observed that MSC-spheroid administration fully normalized both the ethanol-induced astrocyte changes and the GSSG/GSH elevation in the hippocampus, suggesting a potent anti-inflammatory effect of MSC-spheroids. Furthermore, the ethanol-induced increase in MCP-1 expression was also normalized by MSC-spheroid administration. In line with these results, a recent study, which employed siRNA to target TLR4/MCP-1 demonstrated that blocking the activity of TLR4/MCP-1 blunted binge drinking^55^.

The inhibitory effect on ethanol intake observed after the ICV administration of MSC-spheroids in this study was similar to that reported after the ICV administration of human 2D-MSCs (rather than spheroids) that had been activated *in vitro* by incubation with the pro-inflammatory cytokines TNFα and IFNγ^19^. Intra-cerebroventricularly injected rat MSCs without prior *in vitro* activation were also shown to markedly inhibit relapse-like drinking in rats^18^. While these early findings constituted a significant proof-of-principle, the main disadvantage of the ICV administration is its invasiveness. Thus, the possibility of intravenously administering a novel form of 3D-MSCs, namely MSC-spheroids constitutes a significant translational advantage. The effect on biochemical and histological parameters of an ICV injection of 2D-cultured MSC^19^ was remarkably similar to data presented here for the ICV administration of 3D MSC-spheroids.

Several studies have demonstrated that when MSC-spheroids are disaggregated, the cells obtained show a significant size reduction, on the order of 0.25–0.4 the volume of their conventional 2D-cultured counterparts^15,27^. This size reduction (to the size of leukocytes) increases the number of cells that reach the brain after an intravenous administration. As was observed, due to their large size, when 2D-cultured MSCs were intravenously injected, the cells were mostly entrapped in pulmonary capillaries, with few cells reaching the brain. Conversely, when spheroid-derived MSCs were intravenously injected, fewer cells were entrapped in the lungs and more reached the brain, where MSC-spheroids could be detected adhered to brain blood vessels, and were also present in the brain parenchyma. These data agree with studies of Ge *et al*. who described that the intra-carotid injection of conventional 2D-cultured MSCs caused severe vascular obstruction, compared to the lack of dysfunction induced by 3D-cultured MSCs^56^.

Surprisingly, we observed that the administration of MSC-spheroids by the non-invasive intravenous route, while distributed in a large volume, induced strong and long-lasting reductions in chronic alcohol intake and in relapse-like drinking that were almost identical to those observed after the ICV administration of MSC-spheroids. The inhibitory effects of MSC-spheroids on chronic ethanol intake by either route was nearly maximal within 24-hours of their administration, suggesting that the release of soluble factors is associated with their inhibitory effects.

While we cannot discard that part of the effect of MSC-spheroids on ethanol intake and astrocyte morphology may in part have a hepatic origin, several studies have shown that MSCs hold their natural potential to home to the brain in injured sites^57,58^. However, the precise localization of the infused cells in brain areas is not known. In alcoholic rats, one possibility is that increased permeability of the blood-brain barrier induced by ethanol^59^ allows for selective cell entry to the brain.

Safety considerations are a main concern when using a systemic route of stem cells administration. Available results from clinical studies support the overall safety of cell therapy using MSCs by the intravenous route^60,61^ since these cells are non-immunogenic nor associated to tumor formation. The present study indicates that the intravenous administration of MSC-spheroids did not induce changes in classical signs of sickness (reduced weigh gain, reduced fluid intake) compared with vehicle treated rats, nor differences in specific markers such as the presence of TUNEL positive cells in the brain or motor coordination and open field performance. Since we detected MSCs also in liver and kidneys we measured biochemical parameters characteristic of liver damage, including serum transaminases AST and ALT, markers of hepatic function such as serum bilirubin and markers of renal function such as creatinine and nitrogen ureic levels. No differences were detected in any of these markers between the alcoholic animals treated with MSC-spheroids or with vehicle, suggesting that the intravenous administration of MSCs-spheroids is safe.

Neuroinflammation and astrocyte activation have been implicated in drug-use disorders^62^, as well as in a variety of other brain diseases, including Alzheimer’s disease, Parkinson’s disease, Huntington’s disease, multiple sclerosis and amyotrophic lateral sclerosis, conditions in which the potential of MSC therapy has been reported^38,63,64^. The present study broadens the therapeutic potential of MSC-spheroids to alcohol-use disorders, in which available therapies have limited effectiveness; in the range of 15 to 20% (reviewed by Donohue *et al*.)^65^.

Overall, the study shows that a single dose of mesenchymal stem cell-spheroids administered intravenously or intracerebrally to rats consuming alcohol chronically fully abolished alcohol-induced neuroinflammation, inhibited voluntary alcohol intake and reduced relapse-like binge drinking by 80–90%, with significant effects that lasted at least 3 to 5 weeks. Intravenously administered mesenchymal stem cell-spheroids may constitute a novel treatment for alcohol-use disorders.

## Methods

### Isolation, expansion and characterization of human adipose-derived mesenchymal stem cells (hAD-MSCs)

Human AD-MSCs were isolated from fresh subcutaneous adipose tissue obtained from aspirates of four patients undergoing cosmetic liposuction at Clínica Alemana, Santiago, Chile after obtaining written informed consent. Protocols were approved by the Ethics Committee of Facultad de Medicina, Clínica Alemana-Universidad del Desarrollo. AD-MSCs were isolated and expanded as previously described^66^. All methods were performed in accordance with the internationals guidelines and regulations. Their adipogenic and osteogenic differentiation potential and their immune-phenotyping were determined as previously described^66^ (Supplementary Figure 1).

### Generation and dissociation of MSC-spheroids

Human AD-MSCs at passage three were collected by trypsin digestion and resuspended at a cell density of 7.5 × 10^5^ cells/ml in α-MEM (Gibco, Grand Island, NY) supplemented with 10% fetal bovine serum (FBS) (Gibco, Auckland, NZ). Thirty-five microliters of cell solution per drop (containing 20,000 cells) were seeded onto the cover of a culture plate. Cell drops were cultured inversely for three days as previously reported^15^. To dissociate spheroids, they were digested with 0.25% trypsin/EDTA (Gibco) for 10 min and trypsin was then inactivated by adding an equal volume of saline containing 10% rat serum.

### Measurement of cell size

The sizes of MSCs derived from conventional 2D-MSC cultures or from dissociated 3D-MSC-spheroids were determined by hemocytometer microscopy and flow cytometry. For flow cytometric analysis of cell size, 1 × 10^5^ MSCs were stained for the Annexin V-FITC apoptosis detection kit (Invitrogen, Grand Island, NY) and cell size was estimated from the viable population by comparing forward scatter properties of the cells.

### Quantification of mRNA levels of anti-inflammatory factors in 2D-MSCs and 3D-MSC-spheroids

Three days after spheroid formation, total RNA was purified using Trizol (Invitrogen). RNA isolated from MSCs seeded in conventional 2D-cultures was used as control. One microgram of total RNA was used to perform reverse transcription with MMLV reverse transcriptase (Invitrogen) and oligo dT primers. Real-time PCR reactions were performed to amplify the anti-inflammatory factors IL4, IL5, IL10 and TSG6 using a Light-Cycler 1.5 thermocycler (Roche, Indianapolis, IN) as previously reported^66^. Relative quantifications were performed by the ΔΔCT method.

### Quantification of protein levels of anti-inflammatory factors in the secretome of MSC-spheroids

Three days after MSC-spheroid formation, the secretome was obtained by harvesting the culture media. The media was centrifuged at 400 × g for 10 min to remove whole cells and at 12,000 × g for 20 min to remove cell debris. As control, secretome of the same number of 2D-cultured MSCs was obtained and concentrated using 3 kDa cutoff filters (Millipore, Midlesex, MA) to reach to the same volume of MSC-spheroid secretome. IL10 and TSG6 abundance were evaluated in the secretomes using the human IL10 High sensitivity ELISA kit (Invitrogen) and the human TSG6 ELISA kit (RayBio, Norcross, GA) respectively.

### Animal model of chronic alcohol consumption

Two-month-old female Wistar-derived rats selectively bred as alcohol consumers (University of Chile Bibulous; UChB)^67^ were used in the experiments. It is noted that the present work only studied female rats. For rats selectively bred for their high-ethanol intake e.g. the Indianapolis bred rats, females have or higher (HAD-2) or equal (HAD-1) ethanol intake than males^68^. For Sardinian high-ethanol intake bred rats, females show higher levels of ethanol intake than males^69^. Males of the UChB rat line show a 20% lower ethanol intake than UChB females (unpublished). Unlike commercial strains, rats bred for their high ethanol intake spontaneously consume 10 and 20% ethanol solutions and show relapse-like characteristics seen in alcohol-use disorders. Both the Indianapolis-bred Wistar-derived P-rat (high Preference) and the Santiago-bred Wistar derived UChB (Bbibulous) rat have been shown to markedly increase their ethanol intake following chronic ethanol administration and relapse-like intoxication following by a prolonged alcohol deprivation period followed by re-access to ethanol^17,49^.

Animal experimental procedures were approved by the Ethics Committee for Studies with Laboratory Animals at the Faculty of Medicine, University of Chile (17038-MED-UCH). All methods were performed in accordance with the relevant guidelines and regulations. For induction of chronic alcohol intake, animals had continuous 24 hour free-choice access to 10% v/v ethanol and water for 12 to 17 weeks. Relapse-like alcohol drinking was assessed after the animals were deprived of ethanol for 14 days and were allowed re-access to ethanol solutions for only 60 minutes.

### Intracerebroventricular (ICV) administration of MSC-spheroids

After 100 or 117 days of chronic alcohol consumption, rats were ICV injected with 10 μl of a solution containing 5 × 10^5^ MSC-spheroids resuspended in saline containing 10% rat serum as previously described^19^. Control animals were ICV injected with 10 μl of saline containing 10% rat serum (vehicle).

### Intravenous administration of MSC-spheroids

After 82 days of chronic alcohol consumption, rats under moderate sedation with acepromazine (1 mg/kg i.p.) were slowly injected by the tail vein with 1 × 10^6^ MSC-spheroids in 300 μl of saline containing 10% rat serum. Control animals received 300 μl of vehicle. It is noted that a larger dose of MSC-spheroids was used when administered intravenously, as the volume of distribution by the intravenous route is at least one order of magnitude larger than the volume of distribution of the ICV route.

### A note on the use of vehicle as control

The election of a “mock cell” as control is not simple; even the administration of platelets was shown to contribute to tissue regeneration^70^. Fibroblasts, as an option, are specifically not an “innocuous” control cell, since there is abundant bibliography reporting on the effects associated with the administration of these cells. For example, the transplantation of human MSCs in a rat model or Parkinson’s disease resulted in therapeutic effects while control animals infused with saline showed no effects, and animals injected with fibroblasts presented exacerbated neurodegeneration and motor deficits^71^. Thus, fibroblast administration or the administration of other cells is a research question *per se*. In line with many other research groups, in different animal models^64,72,73^ we have adhered to administering the vehicle carrying the MSCs as control group.

### Determination of chronic daily ethanol intake and quantification of ethanol intake in the alcohol deprivation effect (ADE) relapse model

Ethanol intake was determined every 24 hours from volume difference of inverted graduated cylinders. Ethanol intake was expressed as g of ethanol consumed/kg body weight/day. Seven days after ICV administration or 25 days after intravenous administration of MSC-spheroids a 14-day withdrawal period was imposed, followed by a 60-minute ethanol re-access^19^. Ethanol intake was expressed as g of ethanol consumed/kg body weight/60 minutes.

### Blood ethanol and glutathione determinations

After animals had 60 minutes of re-access to ethanol and water, 100 μl of blood was collected from the tip of the tail. Samples were immediately added to 0.9 ml of distilled water at 4 °C in a glass vial sealed and analyzed by head space gas chromatography (Perkin Elmer SRI 8610) as previously described^74^. The ratio of oxidized and reduced glutathione (GSSG/GSH) was determined in hippocampus samples as previously described^19^.

### Evaluation of MSC-spheroids distribution after intravenous administration

For MSC detection, cells were transduced for 8 hours with a lentiviral vector (MOI 5) coding for GFP as previously reported^75^. 72 hours after transduction, GFP expression was confirmed by confocal microscopy and flow cytometry (Supplementary Figure 2). GFP-MSCs were seeded in conventional 2D cultures or in 3D-spheroids for three days. After that, 2D cultured GFP-MSCs and GFP-MSC-spheroids were trypsinized and labeled with DiR tracer (1,1′-Dioctadecyl-3,3,3′,3′-Tetramethyl indo tricarbocyanine Iodide, Thermo Fisher) according to supplier’s instructions. After labeling, cells were rinsed and slowly injected by the tail vein (1 × 10^6^ 2D-MSCs or 1 × 10^6^ MSC-spheroids in 300 μl of saline containing 10% rat serum). Control animals received 300 μl of vehicle. Twenty-four hours after injection, animals were anesthetized and perfused intracardially with 100 ml of 0.1 M PBS (pH 7.4), followed by 200 ml formalin solution. The brain, lungs, liver and kidneys were removed and DiR fluorescence was inspected in an *In-Vivo* MS FX PRO equipment, **(***In-Vivo* Imaging Systems,Bruker, Ettlingen GE). For GFP detection, brain coronal sections (30 μm) were obtained, counterstained with 4,6 diamino-2-phenylindiol (DAPI, Invitrogen), and examined by confocal microscopy (Olympus-fv10i) at 10 and 60× magnification.

### Evaluation of behavioral parameters

Exploratory behavior was examined in a circular arena (open field) placed in a noise-free room as previously described^76^. Motor coordination was evaluated 24 hour after alcohol deprivation using the rotarod test. The rod accelerated from five rpm to 40 rpm over a five-minute period.

### Evaluation of physiological and biochemical parameters

Water intake was determined daily and body weight determined twice a week. At the end of the experiments, animals were anesthetized with chloral hydrate (280 mg/kg i.p.) and blood samples were obtained by heart puncture. Plasmatic levels of aspartate aminotransferase (AST) and alanine aminotransferase (ALT) (markers of hepatic damage); total and direct bilirubin (markers of hepatic function) and creatinine and blood urea nitrogen (markers of renal function) were evaluated spectrophotometrically using the AST Assay Kit, ALT Assay Kit, Bilirrubin Total Assay Kit, Direct Billirubin Assay Kit (all from Wiener Lab, Argentina), Creatinine Assay Kit (Crystal Chem) and QuantiChrom Urea Assay Kit (BioAssay Systems, Hayward, CA) respectively, following manufacturer´s instructions.

### Determination of astrocyte reactivity

Astrocyte reactivity was determined in hipoccampus as it is the area showing the greatest damage in alcohol chronically fed rats^51,52^. Immunofluorescence against the astrocyte marker glial fibrillary acidic protein (GFAP) was evaluated in coronal cryo-sections of the hippocampus (30 μm thick) as previously reported^19^. Nuclei were counterstained with DAPI. Microphotographs (3 to 4) were taken from the *lacunosum moleculare stratum* of hippocampus using a confocal microscope (Olympus FV10i). The area analyzed for each stack was 0.04 mm^2^ and the thickness (Z axis) was measured for each case. The total length and thickness of astrocyte primary processes was assessed for six GFAP positive cells per Z-stack, using FIJI image analysis software (http://fiji.sc/Fiji) as previously reported^19^.

### Quantification of mRNA levels of MCP-1 in hippocampus

Total RNA from hippocampus of MSC-spheroids or vehicle-treated rats was purified using Trizol. RT-PCR was performed for amplification of the pro-inflammatory chemokine MCP-1 as previously indicated^66^.

### Quantification of GLT-1 levels in prefrontal cortex and nucleus accumbens

Samples of prefrontal cortex and nucleus accumbens of MSC-spheroids or vehicle-treated rats were procured and proteins were extracted. Western blot procedures to examine the levels of GLT-1 were performed as previously described^13^. The same membranes were also assessed for β-actin immunobloting as loading control. Immunoblots were quantified using the Image Studio Lite software. GLT-1 was determined in prefrontal cortex and nucleus accumbens since the levels of GLT-1 in these two areas modulate chronic alcohol intake, as shown in the Indianapolis-bred high ethanol drinker Wistar-derived P-rat^13^.

### Quantification of apoptotic rate

Coronal cryo-sections of the brains (30 μm thick) were obtained and mounted on silanized slides. Presence of apoptotic cells was evaluated by the TUNEL technique, using the *In Situ* Cell Death Detection Kit (Roche) following manufacturer´s instructions. Nuclei were counterstaining with DAPI and samples were observed by confocal microscopy focusing on the hippocampus and quantified using the FIJI image analysis software. The data are expressed as percentage of TUNEL positive cells/DAPI/mm^2^.

### Statistical analysis

Statistical analyses were performed using GraphPad Prism (SanDiego, CA). Data are expressed as means ± SEM. Two-way (treatment × day) analysis of variance (ANOVA), followed by Bonferroni’s post hoc test or ANOVA for repeated measures when required, was conducted to compare the ethanol intake or water intake between vehicle and hMSCs groups. One-way ANOVA followed by Bonferroni’s post hoc test was used to analyze total length and thickness of primary processes of GFAP positive astrocytes for GSSG/GSH ratios, MPC-1 and GLT-1 data. When appropriate, a Student’s t-test was used to determine if two sets of data were significantly different from each other. A level of P < 0.05 was considered for statistical significance.

## Electronic supplementary material

Supplementary Information
