# Supplementary material for: Intravenous administration of anti-inflammatory mesenchymal stem cell spheroids reduces chronic alcohol intake and abolishes binge-drinking
Source: Sci Rep. 2018 Mar 22;8:4325. doi: 10.1038/s41598-018-22750-7 (PMC5864829; doi:10.1038/s41598-018-22750-7)
Supplement: Supplementary file 1 — Supplementary Information [file 41598_2018_22750_MOESM1_ESM.pdf]

SUPPLEMENTARY MATERIALS

Intravenous administration of anti-inflammatory mesenchymal stem cell spheroids reduces chronic alcohol intake and abolishes binge-drinking.

Fernando Ezquer*, Paola Morales*, María Elena Quintanilla, Daniela Santapau, Carolyne Lespay-Rebolledo, Marcelo Ezquer, Mario Herrera-Marschitz, Yedy Israel.

SUPPLEMENTARY FIGURE 1

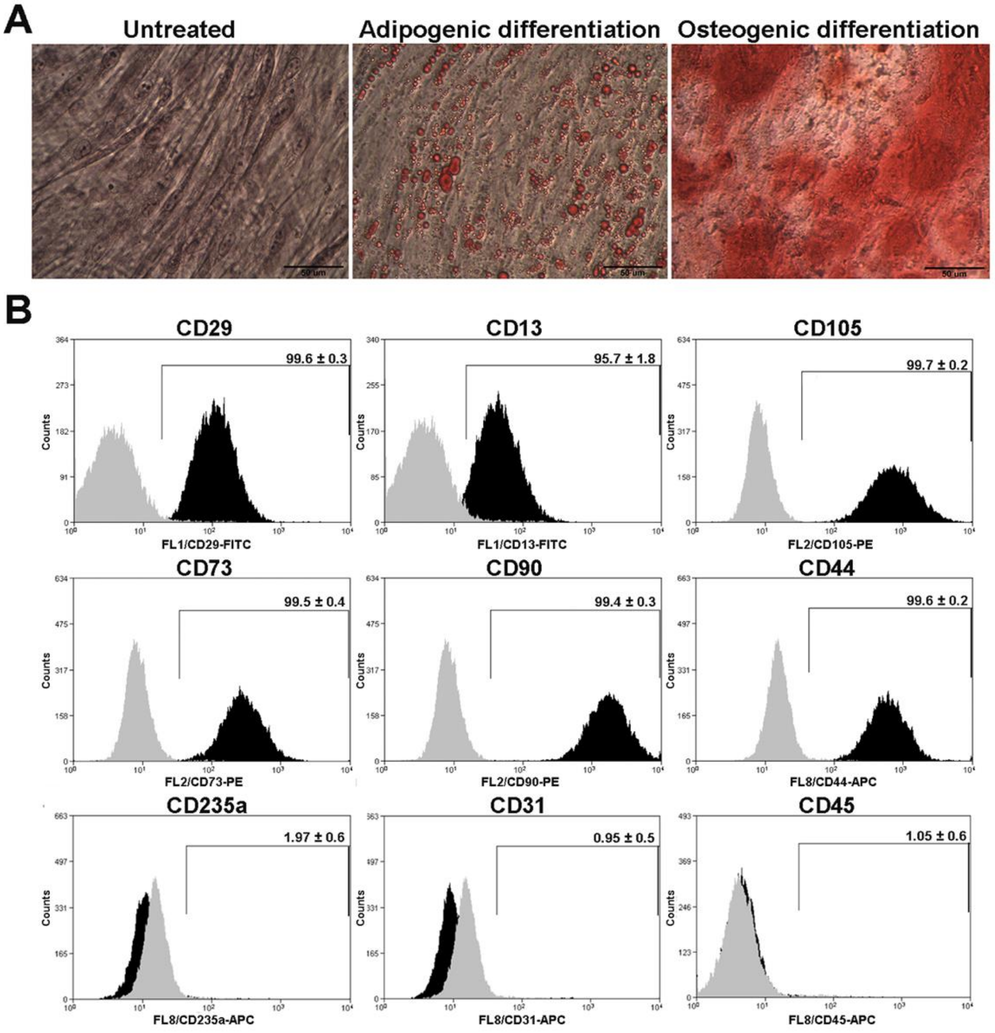

Supplementary Figure 1: Human adipose-derived MSCs display mesenchymal stem cell properties: **(A)** Representative microphotographs of plastic-adherent cells isolated from human adipose tissue differentiated into adipogenic or osteogenic lineages. **(B)** Immunophenotypification of adherent cells according to the expression of putative MSC markers (CD29, CD13, CD105, CD73, CD90, CD44) and the non-expression of markers characteristics of other cell lineages (CD235a, CD31, CD45). Black histograms represent cells labeled with specific antibodies; grey histograms represent cells stained with isotype control antibodies. Data are representative of cells isolated from four donors.

SUPPLEMENTARY FIGURE 2

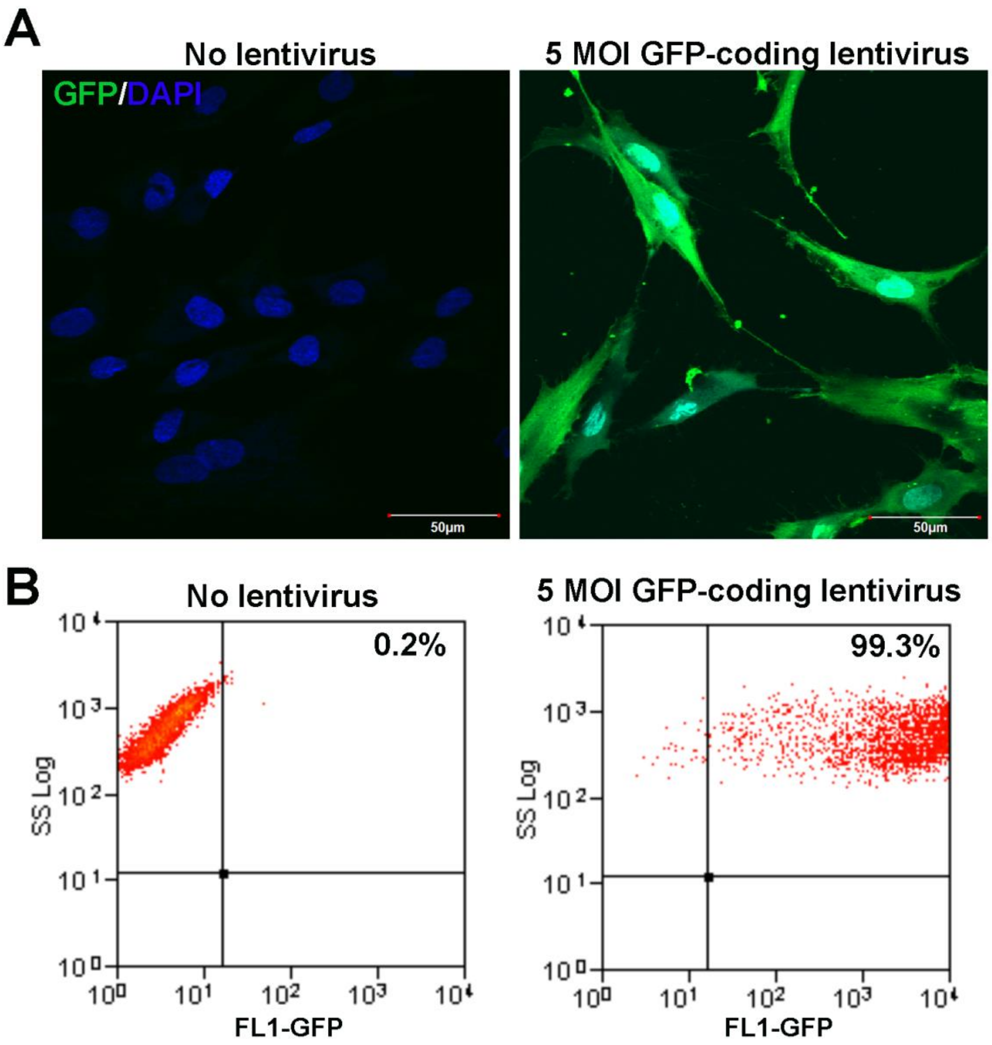

Supplementary Figure 2: Successful transduction of MSCs with lentiviral vector coding green fluorescent protein (GFP): (A) Confocal microscopy microphotographs of GFP expression in MSCs 72 hours after transduction with a lentiviral vector (MOI 5) coding for GFP**. (B)** Flow cytometry quantification of GFP expression in MSCs, 72 hours after their transduction. Data are representative of four transduction experiments.

SUPPLEMENTARY TABLE 1

	Naive	Ethanol+Vehicle	Ethanol+MSC-spheroid
**AST (U/l)**	81.96 ± 12.21	218.57 ± 72.55	190.70 ± 38.03
**ALT (U/l)**	42.53 ± 3.51	90.09 ± 29.68	63.78 ± 8.90
**Direct Bilirubin (mg/l)**	0.28 ± 0.07	0.16 ± 0.08	0.41 ± 0.15
**Total Bilirubin (mg/l)**	2.77 ± 0.98	1.22 ± 0.48	2.28 ± 1.06
**Creatinine (mg/l)**	12.32 ± 2.51	17.2 ± 5.43	14.43 ± 2.57
**BUN (mg/dl)**	42.28 ± 3.93	65.16 ± 9.63	64.63 ± 5.68

Supplementary Table 1: Intravenous administration of MSC-spheroids did not alter biochemical parameters in chronically alcohol ingesting animals.

No differences were observed between the experimental groups in plasma levels of markers associated with hepatic damage (AST and ALT), hepatic function (direct bilirubin and total bilirubin) and renal function (creatinine and BUN). Samples were analyzed forty days after intravenous administration of MSC-spheroids or vehicle. Data are shown as mean ± SEM. N=6 per experimental condition.
